# Primary Atrophic Rhinitis: A Clinical Profile, Microbiological and Radiological Study

**DOI:** 10.5402/2012/404075

**Published:** 2012-11-19

**Authors:** Sampan S. Bist, Manisha Bisht, Jagdish P. Purohit

**Affiliations:** ^1^Department of ENT & Pharmacology, Himalayan Institute of Medical Sciences, Dehradun 248140, India; ^2^Department of ENT, MLB Medical College, Jhansi 284128, India

## Abstract

*Background*. The objective of this prospective study was to evaluate the clinical profile, microbiological flora and radiological features in primary atrophic rhinitis patients and to identify their association with the etiology of primary atrophic rhinitis. *Study design*. Prospective case study. *Materials and methods*. Patients with primary atrophic rhinitis over a two years period were included in the study. Complete blood count, total protein and microbiological analysis from nasal swab were done to evaluate iron deficiency anemia, nutritional status and identification of the pathogenic bacteria respectively. Radiological evaluation was done to study the radiological features of primary atrophic rhinitis. *Observations*. Ninety cases of primary atrophic rhinitis were studied. The most common symptom was nasal crusting. Nasal crust, odour and atrophy of mucosa were the most consistent finding. Nasal myiasis was found in 26.6% cases. The nasal mucociliary clearance time was markedly increased. On investigation there were low value of hemoglobin and total protein in 46.6% and 25.5% patients, respectively. Pseudomonas aeruginosa (37%) was the commonest organism isolated from culture. On radiological evaluation evidence of different grade of sinusitis was seen in 87.7% case. *Conclusion*. The present study suggested that certain bacterial infections, anemia, poor nutrition and hereditary factor may contribute significantly to the etiology of primary atrophic rhinitis.

## 1. Introduction

Primary atrophic rhinitis or ozaena is a well-known disease for ages and was first described by Fraenkel in the latter part of the nineteenth century [1]. The disease is characterized by a sclerotic change in the mucous membrane and abnormal patency of the nasal passages due to atrophic changes in the mucosa and underlying bones, along with thick viscid secretions which, when dry, emit a characteristic foul smell. Atrophic rhinitis can be classified into two types, that is, a primary or idiopathic type where the etiology is not known and a secondary type where the disease develops secondary to some other primary disease. The condition is predominantly seen in young and middle aged adults, especially females (F : M = 5.6 : 1) [2]. Its prevalence varies in different regions of the world. It is a common condition in tropical countries such as India. In the countries with higher prevalence, primary atrophic rhinitis can affect 0.3%–1.0% of the population [3]. The exact etiology of primary atrophic rhinitis is unknown though many theories and hypotheses have been postulated for explanation of atrophic rhinitis. The factors blamed for its genesis are specific infections, autoimmunity, chronic sinus infection, hormonal imbalance, poor nutritional status, heredity, and iron deficiency anemia. Chronic bacterial infection of the nose or sinus may be one of the causes of primary atrophic rhinitis [4, 5]. Classically, Klebsiella ozaenae has been implicated most frequently [2], but other infectious agents associated with atrophic rhinitis include Coccobacillus foetidus ozaenae, Bacillus mucosus, Diphtheroids bacillus, Bacillus pertussis, Haemophilus influenzae, Pseudomonas aeruginosa, and Proteus species. Though it is still not clear whether these bacteria cause the disease or are merely secondary invaders, it may be possible that superinfection with mixed flora causes ciliostasis leading to epithelial destruction and progressive mucosal changes. Nutritional deficiency, especially of iron, fat soluble vitamins, and proteins, has also been suggested in the etiology of primary atrophic rhinitis [6–8]. It appears to be more common in lower socioeconomic classes and those living in poor hygienic conditions [7]. An environmental influence is suggested by its enhanced prevalence in rural areas (69.6%) and amongst industrial workers (43.5%) [2]. It is seen to have a polygenic inheritance in 15%–30% of cases, while other studies have revealed either an autosomal dominant (67%) or autosomal recessive penetrance (33%) [9]. Out of the various proposed etiologies, the theory of chronic persistent infection and autoimmunity has the largest supporters. A diagnosis of primary atrophic rhinitis is essentially clinical and based on a triad of characteristics: foetor, greenish crusts, and roomy nasal cavities. Such a full blown clinical picture is usually seen during later stages and the early course of disease may consist of cacosmia only, with the presence of thick nasal crusts. The objective of this prospective study was to evaluate the clinical profile, microbiological flora, and radiological features in primary atrophic rhinitis patients and to identify their association with the etiology of primary atrophic rhinitis.

## 2. Materials and Methods

This prospective study was carried out in the department of otorhinolaryngology in a tertiary care teaching hospital over a period of two years. A total of 90 cases diagnosed as primary atrophic rhinitis comprised the study cohort. Patients with history of previous nasal surgeries, nasal trauma, and features suggestive of systemic diseases known to affect nasal mucosa leading to secondary atrophic rhinitis were excluded from the study. Prior approval from the institute ethics and research committee and informed consent from the patients were taken. Complete history and clinical finding were recorded in all the cases. Mucociliary clearance test using saccharin as described by Anderson et al. [10] was performed in order to confirm the impaired mucociliary function claimed to be the characteristic of this disease and compare it to the control group. The following investigations were performed to characterize the disease profile: complete blood count to look for iron deficiency anemia, total protein to check for nutrition status, bacterial culture from nasal crust, or discharge for identification of the pathogenic bacteria. Plain X-ray paranasal sinus (occipitomental view) and computerized tomography of the nose and paranasal sinuses were done in selected cases due to financial constrain to study the radiological features of primary atrophic rhinitis and to record concomitant sinus infection. The data was expressed as mean ± SD. The statistics were calculated by the SPSS (Statistical Packages for the Social Sciences) program. 

## 3. Observations 

**Figure 1:**
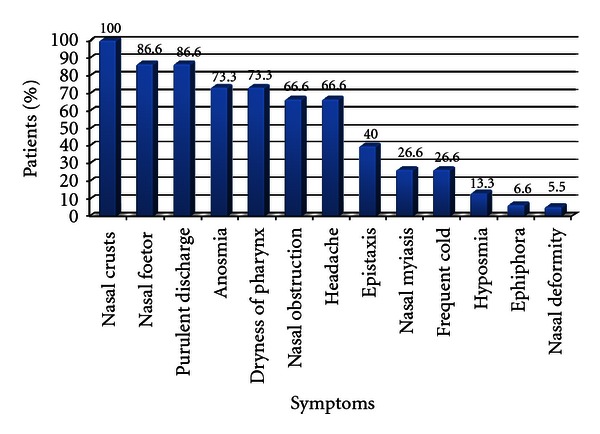
Symptomatology of primary atrophic rhinitis patients (*N* = 90).

**Figure 2:**
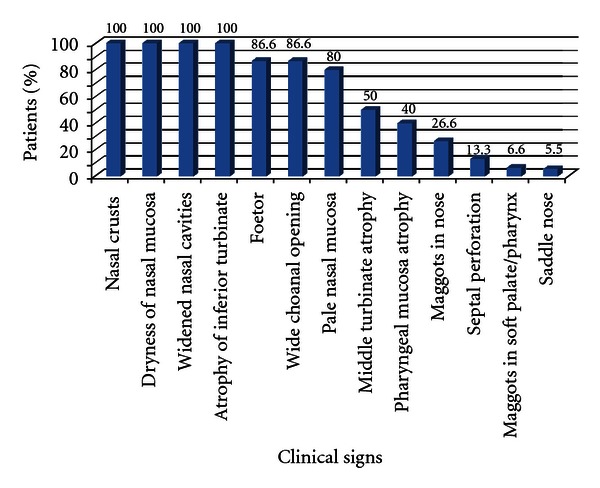
Clinical profile of primary atrophic rhinitis patients (*N* = 90).

90 new patients diagnosed with primary atrophic rhinitis were included in the study. The incidence of primary atrophic rhinitis in the department of otorhinolaryngology was 0.64%. The youngest patient was a 12-year female and the oldest was a 70-year male. The maximum numbers of cases were in 3rd and 4th decades followed by 2nd decade. The male-female ratio was 1 : 2.5. Rural urban population ratio was 2.75 : 1. Low socioeconomic class comprised of 72.2% cases and 27.8% were from middle class while none from high class. Concerning the occupation, majority of patients were housewives (53%), followed by farmer (33.3%) and students (13.3%). Positive family history was found in 12 cases (13%). Out of 12 patients, evidence of hereditary factors could be confirmed in 9 cases by clinical examination. In 8 cases, there was involvement of more than one family member. In all the cases with positive family history the onset of disease was early. The duration of illness of the patients is shown in Table 1. The mean duration of symptoms was 7 years (range from 1 year to 54 years). The details of the presenting symptoms are listed in Figure 1. On examination of nasal cavity, various features noted in study are listed in Figure 2. The severity of the disease was classified into 3 stages, that is, early, advanced, and late advanced, according to the findings in the nasal cavity as recommended by Ssali (1973) [11]. This classification and the number of patients in each stage are shown in Table 2. The majority of patients 54 (59.9%) were in the advanced stage. The mucociliary function was tested in 90 primary atrophic rhinitis patients and in 50 normal control subjects using saccharine; the result is shown in Table 3. The mean value of nasal mucociliary clearance in control group was 9.92 ± 2.25 (mean ± SD) minutes, whereas in primary atrophic rhinitis it was 42.82 ± 11.52 (mean ± SD) minutes. The difference between the two samples was statistically significant (*P* value < 0.0001). 34 (37.7%) cases did not experience sweet taste after 60 minutes which was the maximum observation period for this test.

**Figure 3:**
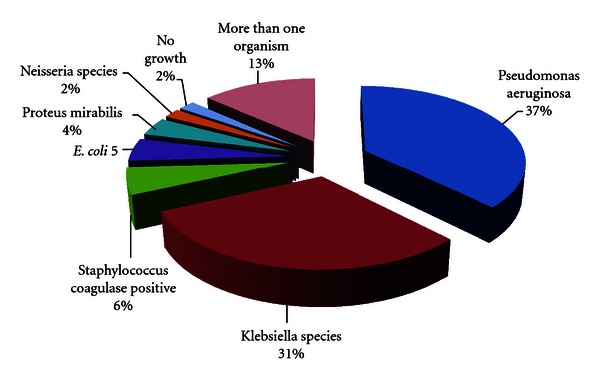
Microbiological organisms isolated from nasal swab in primary atrophic rhinitis patients (*N* = 90).

Table 4 shows the average values of hemoglobin, hematocrit, and total protein in study group of patients. The frequency of variables indicated the number and percentage of patients whose laboratory values were outside the normal range. The hemoglobin level and hematocrit was low in 42 (46.6%) and 37 (41.1%) patients, respectively. In 23 (25.5%) patients' total protein was below the normal range. The results of microbiological study are shown in Figure 3. Pseudomonas aeruginosa was isolated from cultures in 39 (37%) of the patients followed by Klebsiella species 32 (31%). The susceptibilities of Pseudomonas aeruginosa to oral antimicrobial agents were 10%, 57%, and 70% to first, second, and third generation cephalosporins, respectively, and 62% susceptibility to quinolone. Klebsiella species showed 4%, 55%, and 64% susceptibility to first, second, and third generation cephalosporins, respectively. It also showed 62% susceptibility to quinolone and 40% susceptibility to amoxycillin plus clavulanic acid. The cases reported had not received antibiotic treatment over the two weeks prior to presentation. The evaluation of the nose and paranasal sinuses from plain X-rays in 54 (60%) and computerized tomography in 36 (40%) patients is shown in Table 5. Evidence of different grades of sinusitis was seen in 79 (87.7%) patients. Using the level system to describe the degree of sinus involvement in CT scan by Van der Veken et al. [12], CT scan staging was classified from Grade 0 = no change to Grade IV = total opacity. There were maximum numbers of patients in Grade III 31 (34.4%). The most commonly affected sinus was the maxillary 73 (81.1%), followed by ethmoid sinuses in 66 (73.3%). 

## 4. Discussion 

Nearly one and a half century ago, in 1876 Fraenkel first described this chronic distressing condition, incurable yet not fatal. In spite of tireless efforts, the code of its etiology still remains unexplained. Many theories and hypotheses are put forth to explain this condition but have failed to catch general acceptance. Primary atrophic rhinitis is still a prevalent disease in India; the reported prevalence of primary atrophic rhinitis ranges from 0.3 to 1 percent of the population in those countries with high prevalence [3]. In the present study the incidence was 0.62% among the new outpatients cases. Primary atrophic rhinitis is described as a disease of young subject. Most of the authors believe that the disease usually begins at about the age of puberty. In this series age of the patients ranged from 12 to 70 years. Even in the age group above 20 years the onset of the disease could be definitely taken back to an early age. The age of onset is widely distributed before puberty and during child-bearing period suggesting a possible hormonal influence. It has stated that the disease is more common in females than males. In the present study the female : male ratio was 2.5 : 1. Rural urban population ratio was 2.75 : 1. It seems that less caloric diet, early marriage, poor hygiene, and nonavailability of medical facilities in rural area are few causes why it is more common in rural females. Most of cases in our study belonged to poor socioeconomic group living in poor hygienic conditions and receiving substandard nutrition, these factors may be predisposing factors for the development of disease in these patients.

In the present study the majority of the patients presented with long duration of illness along with sequelae and complications of atrophic rhinitis such as nasal septal perforation, saddle nose deformity, nasal myiasis, chronic dacryocystitis, and atrophic pharyngitis. So it can be concluded that primary atrophic rhinitis is a chronic disabling disease. The alarming symptoms seeking attention of the patients were epistaxis and myiasis of nose. Nasal myiasis is an extremely distressing condition seen in neglected cases of primary atrophic rhinitis, especially in patients of lower socioeconomic status living in poor hygienic conditions. In our study myiasis was seen in 26.6% of patients which were much more than that reported in other studies [2]. The main reason for this increased incidence can be attributed to the fact that it was more common in older age patients who are often neglected in families and often neglect their own hygiene. The putrefied nasal debris and foul smell attract flies of the genus Chrysomya. Since this study was conducted in tertiary care centre, so majority of patients 54 (59.9%) having advanced stage of disease or any complication were referred to our institute. Nasal mucociliary clearance is a defense mechanism of the upper and lower respiratory tract. The vital part of this mechanism is adequate quantity of mucus with appropriate rhinological qualities and adequately functioning cilia, which beat in metachronous fashion towards nasopharynx. Any disturbance in number and movement of cilia and mucus production leads to an altered nasal mucociliary clearance as occurs in primary atrophic rhinitis. In present study when compared to normal subjects there was an obvious, statistically significant delay of the mucociliary transport time in primary atrophic rhinitis patients. This finding simply reflects the degree of squamous change of the nasal ciliated epithelium which is very important for the mucociliary function of the nose and later was proven to be due to ciliostatic effects of Klebsiella ozaenae and some other bacteria [13]. A preliminary study on etiology of atrophic rhinitis in Zunji suggested that open-type stoves using burning wood used for everyday cooking increases the SO_2_ concentration in their living environment and may contribute to the etiology of primary atrophic rhinitis [14]. There are other studies also which support the exposure to phosphorite and apatite dust and industrial irritants as a predisposing factor for primary atrophic rhinitis [2, 15]. In our study also, majority of the patients were housewives living in rural areas where using burning wood is widely used for everyday cooking by females and hence were subjected to chronic exposure to irritants in the environment. However, the other largest groups in our study, namely, farmers and students did not reveal any chronic exposure to the environmental factor. Nutritional deficiency has been marked as one of the etiologic factors of primary atrophic rhinitis by many authors. Some authors consider this to be an iron deficiency disease [6, 16–19]. Fat-soluble vitamin deficiency especially vitamin A is also believed to be a predisposing factor [1, 7]. An interesting study from Poland reported that ozaena is almost absent in the well-developed regions and commonly occurs in the developing and underdeveloped countries where the everyday diet is poor in iron, proteins, and vitamins [8]. However, contradicting this finding, a study from Norway reported a high incidence of iron deficiency anemia without a relatively high incidence of atrophic rhinitis [20]. The study in our patients also showed low hemoglobin in 46.66% (42) and low protein in 23.3% (23) which confirm the significance of a nutritional factor. Hereditary or familial tendency in primary atrophic rhinitis is cited by many authors [17, 21]. The disease may be polygenetic and hence heritable. One interesting study showed that 27.4% of cases displayed an inheritance pattern of which an autosomal dominant pattern was seen in 67% and a recessive trait in the rest [9]. In another study, 20% of cases had more than one member of the family suffering from the same disease [22]. Another study based on genetic analysis of a family affected by ozaena also suggested that genetic factor could drive the chronicity of the inflammatory pattern of preexisting infectious nasal disease [23]. Positive family history was found in 13% (12) of cases in present study. In 8 cases, there was involvement of more than one member of the family by the same disease. This supports that the hereditary factor had a role in etiology of this disease. Chronic bacterial infection of the nose or the sinuses is implicated as a cause of primary atrophic rhinitis [4, 5, 24]. In one bacteriological study in 61 Indonesians with atrophic rhinitis, that is, 71.6% Klebsiella species, 32.8% Pseudomonas aeruginosa and 22.9% Staphylococcus aureus were found to be common pathogenic organisms [25]. Another study from Thailand showed that in 46 patients Klebsiella was recovered from the first swab in 78.3% of the patients, and if the results of the second and third swabs were included, 97.8% yielded Klebsiella species. The most common type of Klebsiella found was Klebsiella ozaena (67.4%), Pseudomonas aeruginosa was the second most common organism found in 34.8%, Pr. mirabilis 10.9%, and Staphylococcus aureus 6.5% [2]. Another microbiological study from Egypt in 14 patients reported Klebsiella species in 65% patients and Pseudomonas aeruginosa 14.2% cases. However, as far as the authors are aware, this was the first study which reported the fungal elements in primary atrophic rhinitis and most commonly aspergillus species were isolated in 93% of cases. They concluded that the persistence of purulent secretion, within the setting of impaired mucociliary clearance, leads to saprophytic fungal colonization which contributes greatly to the clinical picture of disease [26]. In our study, contrary to the other studies pseudomonas was the commonest organism isolated followed by Klebsiella species. Bacterial infection of the nose and sinuses has confirmed the significance of chronic bacterial infection in primary atrophic rhinitis although the role of these infections as a cause of the disease remains controversial. There is however little evidence to suggest these organisms cause the disease; they may be secondary invaders. The etiological role of bacteria can only be confirmed or excluded by reproducible experimental studies in animals. Ciliostasis by K. ozaena has been studied as a mechanism in the pathogenesis of atrophic rhinitis [13]. Klebsiella species and some other bacteria common to acute and chronic sinusitis possess the ability to slow ciliary beating (ciliostasis) and disrupt normal coordinated ciliary activity, thereby impairing the mucociliary clearance leading to persistent infection and probably injury to the ciliated epithelium with progressive mucosal changes [13]. So, they do not appear just as an opportunistic colonizer but could be considered as one of the multifactorial etiologies of primary atrophic rhinitis. The antimicrobial susceptibility of these bacteria is dynamic and should be individually studied because long-term antibiotic use is still recommended as the mainstay of medical therapy for primary atrophic rhinitis [27]. Routine radiography is of limited value and has been replaced largely by computerized tomography scanning. Because of the high incidence of concurrent sinusitis, CT is frequently included in the diagnostic evaluation of atrophic rhinitis. The typical CT changes of atrophic rhinitis as reported by Pace-Balzan et al. [28] are as follows: (1) mucosal thickening of the paranasal sinuses, (2) loss of definition of the ostiomeatal complex secondary to resorption of the ethmoid bulla and uncinate process, (3) hypoplasia of the maxillary sinuses, (4) enlargement of the nasal cavities with erosion and bowing of the lateral nasal wall, and (5) bony resorption and mucosal atrophy of the inferior and middle turbinates. In the present study atrophic change of the mucosa, bone and widening of the nasal cavity and hypoplastic maxillary sinuses were constantly observed and correlated well with the severity of atrophic rhinitis seen clinically. It was reported that these findings occurred late in the disease as a result of the changes in the nose [28]. In one study, 60% of the patients showed thick bony wall and a small cavity of the maxillary sinus which were confirmed on antroscopy [29]. There are other studies in the literature in which there are reports of reduced pneumatization of the paranasal sinuses, in particular of the maxillary and ethmoid sinuses, with thickening of the bony walls [27, 30, 31]. In our study, the absence of the frontal sinus in 27 (30%) cases was detected in addition to the above-mentioned findings. Plain X-rays directly had no role in the diagnosis of primary atrophic rhinitis but are sometimes considered especially prior to a proof puncture. But if a surgeon is contemplating sinonasal surgery for disease clearance, complete CT scanning of nose and sinuses is essential.

Although many factors have been cited previously as the possible cause of primary atrophic rhinitis, it is proposed that the initial trigger for primary atrophic rhinitis is a virulent bacterial infection of the nasal lining, which leads to damage of the ciliated epithelium. This initiates the cascade of events leading to chronic inflammation of the nasal mucosa, with osteomyelitis of the turbinate bone. The common characteristics found in our patients indicate that only bacterial infection, anemia, poor nutrition, and hereditary factor could be one or more of its multifactorial etiology.

## Figures and Tables

**Table 1:** The duration of illness in primary atrophic rhinitis patients (*N* = 90).

Duration of symptoms in years	No. of cases (percentage)
≤1	12 (13.3)
1–5	24 (26.6)
6–10	36 (40.0)
≥10	18 (20.0)

**Table 2:** Severity of disease according to the Ssali classification (*N* = 90).

Staging	Crust	Odour	Atrophy	No. of cases (%)
Early	Minimal	Mild foetor	Only at turbinates	12 (13.3%)
Advanced	Lots	Foul	Generalized, including bones	54 (59.9%)
Late advanced	Extensive	Foul	Ulceration/bleeding, very large nasal cavities	24 (26.6%)

**Table 3:** Comparison of mucocilliary clearance time between normal control and primary atrophic rhinitis patients.

Group	Number	Nasal mucocilliary clearance time range (in minutes)	Mean ± SD
Normal control	50	7.3–14.7	9.92 ± 2.25
Atrophic rhinitis case	90	24–>60	42.82 ± 11.52

*P* value control versus case <0.0001.

**Table 4:** Hemoglobin, hematocrit, and total protein in primary atrophic rhinitis patients and variation from normal range.

Investigation	Normal range	Atrophic rhinitis		Frequency of variables	
		RowSpanEmpty	Range	Mean ± SD	Number	Percentage (%)
Hemoglobin (*n* = 90)	12–18 g/dL	6.5–15.6	11.69 ± 1.88	42	46.66
Hematocrit (*n* = 90)	38–52%	25–48	39.09 ± 5.92	37	41.1
Total protein (*n* = 90)	6–8 g/dL	4.6–8.2	6.31 ± 1.21	23	25.55

**Table 5:** Radiological findings from plain X-rays and CT scan in primary atrophic rhinitis patients (*N* = 90).

Sinusitis		Sinus involved		Other findings	
Grade	Number (%)	Sinus	Number (%)	Findings	Number (%)
Grade 0	11(12.2%)	Maxillary	73 (81.1%)	Atrophy of bone and mucosa	78 (86.6%)
Grade I	4 (4.4%)	Ethmoid	66 (73.3%)	Widening of nasal cavity	77 (85.5%)
Grade II	22 (24.4%)	Frontal	32 (35.5%)	Lateral bowing of the lateral nasal wall	65 (72.2%)
Grade III	31(34.4%)	Sphenoid	17 (18.8%)	Thickness of medial wall of maxillary sinus	62 (68.8%)
Grade IV	22 (24.4%)			Hypoplasia of maxillary sinus	66 (73.3%)
				Absence of frontal sinus	27 (30%)
